# Target-Specific Delivery and Bioavailability of Pharmaceuticals via Janus and Dendrimer Particles

**DOI:** 10.3390/pharmaceutics15061614

**Published:** 2023-05-29

**Authors:** Jaison Jeevanandam, Kei Xian Tan, João Rodrigues, Michael K. Danquah

**Affiliations:** 1 CQM—Centro de Química da Madeira, MMRG, Universidade da Madeira, Campus da Penteada, 9020-105 Funchal, Portugal; jaison.jeevanandam@staff.uma.pt; 2 GenScript Biotech (Singapore) Pte. Ltd., 164, Kallang Way, Solaris@Kallang 164, Singapore 349248, Singapore; kxtan5@gmail.com; 3 Department of Chemical Engineering, University of Tennessee, Chattanooga, TN 37403-2598, USA

**Keywords:** dendrimers, Janus nanoparticles, drug delivery, biocompatibility, nanoformulation, pharmaceuticals

## Abstract

Nanosized Janus and dendrimer particles have emerged as promising nanocarriers for the target-specific delivery and improved bioavailability of pharmaceuticals. Janus particles, with two distinct regions exhibiting different physical and chemical properties, provide a unique platform for the simultaneous delivery of multiple drugs or tissue-specific targeting. Conversely, dendrimers are branched, nanoscale polymers with well-defined surface functionalities that can be designed for improved drug targeting and release. Both Janus particles and dendrimers have demonstrated their potential to improve the solubility and stability of poorly water-soluble drugs, increase the intracellular uptake of drugs, and reduce their toxicity by controlling the release rate. The surface functionalities of these nanocarriers can be tailored to specific targets, such as overexpressed receptors on cancer cells, leading to enhanced drug efficacy The design of these nanocarriers can be optimized by tuning the size, shape, and surface functionalities, among other parameters. The incorporation of Janus and dendrimer particles into composite materials to create hybrid systems for enhancing drug delivery, leveraging the unique properties and functionalities of both materials, can offer promising outcomes. Nanosized Janus and dendrimer particles hold great promise for the delivery and improved bioavailability of pharmaceuticals. Further research is required to optimize these nanocarriers and bring them to the clinical setting to treat various diseases. This article discusses various nanosized Janus and dendrimer particles for target-specific delivery and bioavailability of pharmaceuticals. In addition, the development of Janus-dendrimer hybrid nanoparticles to address some limitations of standalone nanosized Janus and dendrimer particles is discussed.

## 1. Introduction

Nanoparticles have gained considerable attention among researchers as a potential drug delivery system due to their unique properties, such as their high surface-to-volume ratio and surface charge-dependent behavior, compared to their bulk counterparts [1,2,3]. The properties of nanoparticles depend on their size and shape, which can be tailored by selecting an appropriate synthesis approach [4]. Dendrimers, micelles, liposomes, and biopolymers are the most commonly used drug-delivery nanoparticles [5]. Micelles are colloidal suspensions formed by the dispersion of amphiphilic lipid molecules in a liquid and have a hydrophilic head and a hydrophobic tail [6]. Micelles as a drug delivery system have advantages such as improved solubility of highly lipophilic drugs, controlled drug release, the ability to adjust their physiochemical properties, and protection of the drug from environmental factors. However, they have limitations such as low drug-loading capacity, high dependence on critical micelle concentration, and limited applicability to only lipophilic drugs [7]. Liposomes are small artificial spherical vesicles formed using natural, nontoxic phospholipids and cholesterol and have benefits such as biocompatibility and hydrophilic/hydrophobic characteristics [8]. However, liposomes as a drug delivery system face limitations such as high production cost, limited shelf life, vulnerability to oxidation and hydrolysis of phospholipids in certain conditions, instability, fusion, and potential release of encapsulated drugs [9]. Biopolymers (polymers synthesized or extracted from biological source) have also been used for drug formulation, but they often lack solubility or have pH-dependent solubility, which limits their use [10,11].

Dendrimers are synthetic, tree-like hyperbranched polymers with a high number of functional groups and an open molecular structure. They are designed as artificial macromolecules with void spaces for drug storage and targeted release [12,13,14]. However, dendrimers have limitations such as high non-specific toxicity, drawbacks during scale-up experiments, and low hydro-solubility [15]. Despite these limitations, they have potential as nanoparticles for drug delivery. Janus nanoparticles are a recent addition to the range of nanoparticles, featuring the integration of two or more chemically distinct components into a single structure. They possess unique properties based on their synthesis approaches and the materials infused into the Janus structure [16,17,18,19]. However, the complex synthesis process and toxicity due to chemicals involved in the synthesis approach are limitations of Janus nanoparticles [17,20]. The incorporation of Janus and dendrimer into a composite material has been proposed to enhance drug delivery ability and reduce limitations [21,22]. This article discusses various nanosized Janus and dendrimer particles for target-specific delivery and bioavailability of pharmaceuticals. Additionally, the emergence of Janus-dendrimer nanoparticles to overcome the limitations of standalone nanosized Janus and dendrimer particles is discussed.

## 2. Overview of Nanosized Janus and Dendrimer Particles

### 2.1. Janus Nanoparticles

Janus nanoparticles were first discovered by Pierre-Gilles de Gennes, the Nobel Laureate who pioneered fabricating microparticles ‘Janus grains’ with an apolar and polar side [23]. The word ‘Janus’ comes from the two-faced Roman God of gates, which defines Janus nanoparticles as anisotropic particles that possess two different compartments with varying functionalities, material compositions, morphology, size, shape, and biochemical properties. Janus nanoparticles are originally from polymeride but can be subcategorized as organic/polymeric, inorganic, or hybrid of organic and inorganic Janus particles [24,25,26,27]. In addition to the typical spherical shape, Janus nanoparticles can be fabricated into different conformations, which include rod [28], dumbbell [29], platelet [30], and snowman [31,32].

Due to their asymmetric faces, Janus nanoparticles can improve the stability of different phases [33]. This has then broadened their biomedical and clinical applications from emulsion stabilizer, bio-sensing, bio-catalysis, molecular imaging, and diagnostic tools to pharmaceutical targeted drug delivery systems [34], offering significant benefits over the conventional mono-functional particles. This is highly ascribed to the tunable properties of Janus nanoparticles whereby their different surfaces or compartments can be modified with individual functionality. This includes hybrid particles with one amphiphilic surface and another stimuli-responsive surface [19]; Janus nanoparticles made of organic and inorganic compartments [35]; or biocompatible particles [36] for targeted medical treatments.

This enables Janus nanoparticles to be utilized as delivery carriers to carry different drug molecules with the combination of various functionalities. Otherwise, as a delivery system, one hemisphere can load medical drug molecules while another side acts as a targeting element with high specificity toward targeted cells. Janus nanoparticles have practical medical and environmental applications, such as detecting water contaminants and environmental pollutants and serving as superior candidates for cancer theranostics due to their high loading capacity and tunable properties. Janus nanoparticles made of silver/chitosan have also been reported to exhibit high antimicrobial effects against bacteria such as *Escherichia coli*, *Salmonella choleraesuis*, *Bacillus subtilis*, *Staphylococcus aureus*, indicating their potential applications in food sector [37]. Interestingly, there are Janus nanoparticles used to detect DNA and metals for monitoring applications. A streptavidin-modified retroreflective Janus particle can selectively sense the presence of mercury ions with up to 0.027 nM detection limit [38] whilst a hybrid of gold-silver nanorod and polyaniline has also been developed as a Janus nanoparticle, serving as a surface-enhanced Raman scattering sensor for the detection of mercury [39]. In addition, gold-silver Janus nanoparticles have been exploited as aptasensor to detect toxins such as Ochratoxin A quantitatively, which can be widely used in real systems, including red wine monitoring [40]. The above examples highlight the vast potential of Janus nanoparticles for a broad range of applications, offering numerous benefits to various industries.

### 2.2. Dendrimers

The Greek phrase ‘dendron’, which means trees or branches, is the source for the word ‘dendrimer’. Dendrimers are symmetrical, generation-dependent spherical polymers consisting of a core and dendrons (branches), possessing a hyperbranched, three-dimensional structure [13]. In 1941, Paul John Flory and colleagues (Nobel Prize in Chemistry 1974) introduced the theory of highly branched polymers [41,42], which can be synthesized through polycondensation of a monomer with one or more functional groups, avoiding the gelation process [43]. However, it was not stable and are without a cavity. Later, Vogtle and his team (1978) reported the formation of the first non-skid chain-like and cascade-like molecules with the topology of the molecular cavity, which is considered the earliest dendritic polymer form. The term “hyperbranched polymer” was first coined by Kim and Webster in 1988 in reference to the synthesis of soluble hyperbranched polyphenylene. This term was later used to describe the structure of dendrimers [44]. However, these particular types of polymers attract the academy’s attention only with the work of Tomalia et al. (1985) [45] and Newkome et al. (1985) [46]. Further, Tomalia not only coined the term “dendrimer” as made a drastic breakthrough in dendrimers field by forming in a controlled manner using divergent synthesis, poly(amidoamine) (PAMAM) dendrimers with a hollow core in the center and outward branches of tendrils [47]. Currently, there are about 100 dendrimer families, which include beyond poly(amidoamine) (PAMAM) dendrimers, among others, polypropyleneimine (PPI), polyester-, polyamide-, phosphorus, and polyether-based dendrimers [12].

**Figure 1:**
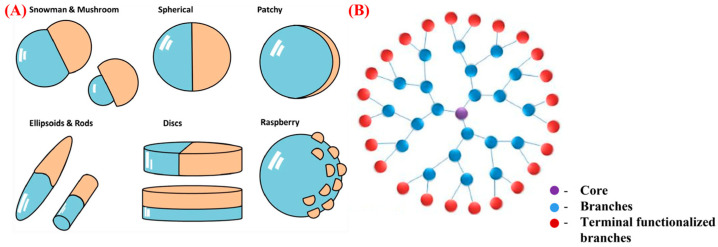
The general structure of (**A**) Janus, Adapted with permission from Honciuc Ref. [62]. Copyright 2019 Springer and (**B**) dendrimer particles, Adapted with permission from Araujo et al. Ref. [63]. Copyright 2018 MDPI.

Dendrimers’ molecular mass and size are specifically controlled during the polymerization process, which is not possible during linear polymer formation [48]. The unique molecular architecture of dendrimers results in improved physical and chemical properties compared to traditional linear polymers [49]. In general, dendrimers have a tightly packed spherical structure with excellent rheological properties and low viscosity than linear polymers [50,51]. It’s worth mentioning that the intrinsic viscosity of a dendrimer reaches its peak at the fourth generation as its molecular mass increases [51,52]. The high solubility, miscibility, and reactivity of dendrimers can be attributed to the multiple chain-ends present in their structure [53]. Similarly, the solubility of the dendrimers depends on their surface group, where dendrimers with hydrophilic and hydrophobic terminations are soluble in both polar and nonpolar solvents, respectively [54]. Furthermore, the spherical shape and presence of internal cavities in dendrimers make them ideal for encapsulating desired molecules or drugs within the macromolecules [55]. These novel polymers are further sub-classified into cationic, neutral and anionic dendrimers, based on their surface charge [56]. It is worth noting that cationic dendrimers are cytotoxic and hemolytic, whereas dendrimers with carboxylate surfaces that are anionic are considered nontoxic for a broad range of concentrations [57,58]. However, the properties of dendrimers are significantly influenced by factors such as pH, solvent, precursor salt, and concentration [59]. Moreover, preparation of dendrimer in the nano-regime will further enhance their properties, due to their exceptional high surface-to-volume ratio and unique structure [60,61]. Figure 1 shows the structural aspects of Janus and dendrimer particles.

## 3. Rational Design and Synthesis Approaches

### 3.1. Janus Nanoparticles

Janus nanoparticles are known as a new generation of smart building blocks for material evolution. They are innovative materials designed to have two or more physical and biochemical anisotropy as well as assembled layers to serve multiple functionalities. Their unique characteristics and structural designs make them superior to conventional particles. Their superior characteristics are such as the high interfacial activity; engineering of their interfacial activity via external stimulus (i.e., pH, temperature, or ionic strength); recyclable for sustainable usage attributed to the possibility of stimuli-induced separation; high surface-to-volume ratio; manipulation of mass transport across different interfacial layer; and their multi-functionalities in one Janus nanoparticle [64]. This allows them to be flexible as either a macro-phase separation or stable emulsion. Furthermore, many materials especially biodegradable or natural materials such as chitosan, alginates, and cellulose, have been widely used for the Janus nanoparticles fabrication [37,65,66,67,68,69]. Janus nanoparticles that respond to changes in pH have been reported for use in bio-imaging and anti-tumor drug delivery. These nanoparticles enable real-time monitoring and tracking of drug release [70,71].

Various synthetic techniques are available and optimized to fabricate Janus nanoparticles with the desired shape, size, and physical or biochemical functionality on their opposite side. Three widely recognized conservative fabrication methods are masking, phase separation, and self-assembly. Masking is the simplest technique used to fabricate Janus nanoparticles by covering up one side using either solid or liquid blocking surfaces while exposing the other side of the particles for chemical modifications. For instance, inorganic Janus particles were produced via the partial coating of calcium carbonate particles with platinum to create non-Brownian movement in the acidic environment of the tumor site [72]. This approach allows scientists to manipulate the chemical and structural features of Janus nanoparticles, resulting in exceptional design and functionality. However, the masking technique is only appropriate for small-scale laboratory production, making it useful for initial investigation and basic testing. The phase separation technique works by mixing two or more incompatible polymers or components before separating them into different compartments of a single Janus nanoparticle. The pH of the aqueous phase, the makeup of the dispersed phase, spreading coefficients, and interfacial tension can often impact phase separation and particle shape [73]. Self-assembly from di-block copolymer is considered one of the easy and simple methods to synthesize different polymeric Janus nanoparticles by adjusting the cross-linking degree and molecular weights or ratio of the blocks used [34].

Pickering emulsion on the other hand utilizes the interface of two immiscible fluids for both particle adsorption and unique surface modifications to synthesize the anisotropic particles. This technique enables the production of emulsions such as silica-based amphiphilic Janus particles with colloidal stability that lasts for more than 3 weeks [74]. Nonetheless, this technique comes with limitations including the tedious multiple steps that require strict monitoring; limited encapsulation and controlled release capacity; unsuitability for biodegradable polymeric Janus particles due to the high temperature and specific solvents used to eliminate the masking layer [75].

Methods such as microfluidic assembly and a ‘lab-on-a-chip’ system have also been exploited to fabricate Janus-like dimer capsules. This technique comprises a series of steps such as fluidic assembly, cross-linkage, and droplet formation that occur on a microfluidic chip without manual control. Lu and team [67] have designed Janus-like dimers whereby the functionality and structure of each lobe have been precisely controlled to resume bowling pin and snowman structures. This method allows the formation of Janus-like dimer capsules by having two individual biopolymer mixtures with distinct compositions from two different channels meet at the junction and fuse into one stable formation with distinct compositions via controlled cross-linking and coalescence. There is also a study reported on the use of a microfluidic-mediated fabrication method to produce Janus nanoparticles in a microchannel whereby two adjacent fluid streams with a laminar flow are separated into discrete droplets via the immiscible phase and finally photo-polymerized into Janus nanoparticles [76]. However, this method comes with challenges such as low output efficiency, precise control, and limited polymer choices. Furthermore, there is a fluidic nanoprecipitation system developed to fabricate Janus nanoparticles made of PLGA. The results showed that these polymeric Janus nanoparticles effectively encapsulated two distinct drugs, Paclitaxel (a hydrophobic drug) and Doxorubicin Hydrochloride (a hydrophilic drug), resulting in varied drug release behavior [77]. This is a single-step method that utilizes dual inlets to deliver each half of the Janus compartment into the precipitation flow.

**Figure 2:**
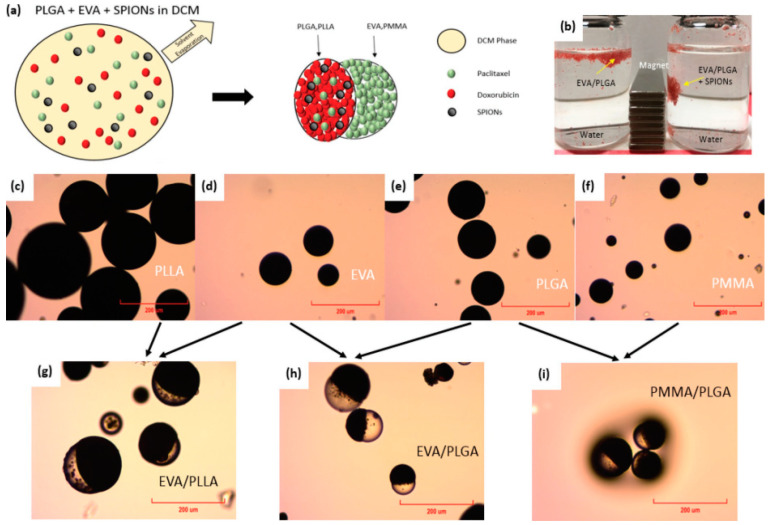
A simple and single-step solvent emulsion technique used to fabricate polymeric Janus particles for theranostic applications. (**a**) Theranostic polymeric Janus particles made of both biodegradable and non-biodegradable polymers, (**b**) magnetic response of drug and SPIONs-encapsulated EVA/PLGA Janus particles, (**c**–**f**) a comparison of SPION encapsulation in different polymers i.e., PLLA, EVA, PLGA, and PMMA, (**g**–**i**) the formation of SPIONs-encapsulated Janus particles: EVA/PLLA, EVA/PLGA, and PMMA/PLGA. Reproduced with permission from Lim et al. Ref. [75]. Copyright 2019 Wiley.

Another team of scientists [75] devised a straightforward, one-step solvent emulsion method for creating hybrid Janus particles. This approach enables the targeted encapsulation of both diagnostic and therapeutic agents into separate compartments of the Janus complex through drug-polymer interactions and the use of specific biopolymer ratios, as illustrated in Figure 2. This technique is capable to offer a more cost-effective, scalable, and seamless approach to synthesizing dual drug-encapsulated theranostic Janus particles with controlled and different release kinetics. With this method, both the immiscible biodegradable and non-biodegradable polymers are emulsified and segregated into two different compartments due to the interfacial tensions. This one synthesis setup technique could replace the single drug-encapsulated particle due to its simplicity and scalability.

### 3.2. Dendrimers

**Figure 3:**
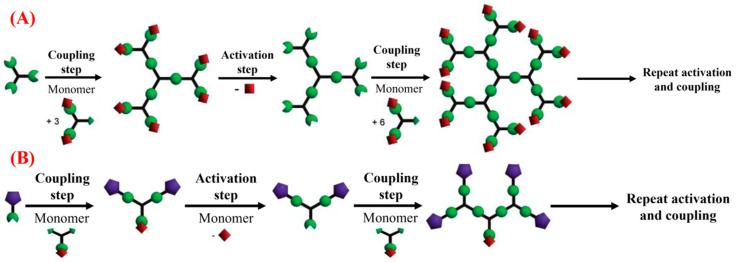
Schematic representation of (**A**) Divergent and (**B**) convergent approach, Concept adapted from Grayson et al. Ref. [85]. Copyright 2001 American Chemical Society.

Generally, dendrimers are synthesized using conventional methods, such as cascade reactions, and divergent as well as convergent approaches [78]. The cascade strategy involves the assembly of three sub-nano/picoscopic-sized repeated units via reaction with a divalent terminus to produce a branched cell with a branch juncture. The generation zero (0) of the dendrimer is formed by attaching the first branch to the reference point for yielding a labeled branch with a valency of four. Later, the first and second generation of dendrimers was formed by stepwise attachment of repeat units according to their incipient valency [79]. Even though the cascade strategy is initially used, convergent and divergent approaches are commonly utilized for the fabrication of dendrimers as shown in Figure 3. In a divergent approach, the dendrimers are synthesized from the core to the branches through repeated coupling and activation steps in an outward direction [45]. Hence, the cascade strategy is also considered a type of divergent approach for dendrimer formation [80]. Recently, Rauch et al. (2020) utilized an iterative divergent approach for the preparation of conjugated starburst borane dendrimers. In this study, the dendrimer was prepared in three steps: the functionalization of iridium-catalyzed carbon-hydrogen borylation and activation of fluorine-generated boronate ester with potassium bi-fluoride and expansion of trifluoroborate salts with aryl Grignard reagent reaction [81]. In a convergent approach, the branches (dendrons) of the dendrimer were prepared initially, which is finally coupled to the moiety in the core after the activation of their focal point [82]. It can be noted that the growth of the dendron is straightforwardly monitored via a convergent method, compared to a divergent approach [83]. Bondareva et al. (2020) prepared sulfonimide-based dendrimers and dendrons with the help of a convergent approach. In this study, both convergent and divergent methods were used to create sulfonimide-based dendrons and dendrimers with chlorosulfonic groups as the central focus. Suprisingly, the results indicated that the convergent method of creating dendrons reached its practical limit in the third generation, making a divergent approach necessary for producing sulfonimide-based dendrimers with more generations [84].

Apart from the conventional divergent and convergent approach, revised traditional methods, such as the hyper monomer method, double-stage convergent growth, and double exponential growth were also used for the synthesis of dendrimers [86]. Monomers with higher functional group numbers were employed in the hyper monomer approach for dendrimer synthesis in fewer steps, compared to the traditional AB_2_ monomer [87]. Balaji and Lewis (2009) synthesized a novel aliphatic polyamide dendrimer by AB_2_ hyper monomer strategy and double exponential growth. The study showed that the yield of the dendrimer is about ~93%, which is reduced to about ~89% after 30 min of the purification process [88]. In a final step, low-generation dendrimers and dendrons are linked together through parallel synthesis in a double-stage convergent growth approach [89]. Recently, Agrahari et al. (2020) demonstrated the synthesis of novel glycol-dendrimers and dendrons coated with galactose was achieved through an efficient click approach and double-stage convergent method. The study emphasized the synthesis of a novel galactose coated 9-peripheral glycodentromer of the zeroth generation and a galactose-coated 27-peripheral glycodentromer of the first generation [90]. Further, low-generation dendrons that were fully protected or deactivated were created, and then actively selected at their periphery or central focus, and joined to produce higher generations of fully protected dendrons through a double exponential growth method [91]. Hartwig et al. (2010) prepared A novel polyglutamate dendrimer of the fourth generation was produced through an iterative binomial exponential growth method. The research demonstrated that this synthesis technique can aid in incorporating either D-alt-L or all-(L) stereochemistry in the peptide backbone and is useful for post-functionalization of the dendritic core and periphery [92].

Additionally, orthogonal, chemoselective reactions (click chemistry), one-pot synthesis, and heterofunctional fabrication approaches were introduced for dendrimer formation. The click approach makes use of Diels-Alder cycloaddition and thiol-ene coupling, which are highly selective chemical reactions, and offer a reliable platform for the synthesis of complex macrostructures [93]. Ma et al. (2009) fabricated a novel polyester dendrimer via a sequential click coupling of asymmetrical monomers. In this study, mechanistic or kinetic chemoselectivity was combined with click reactions between the monomers for efficient dendrimer synthesis via simple sticking of generation by generation together [94]. When different monomers, such as Ab_x_ and CD_y_, instead of AB_n_ monomer in divergent growth approach for dendrimer formation is termed as orthogonal growth method [95]. Liu et al. (2022) fabricated scaffold-modifiable dendrons via an orthogonal protection strategy. The study revealed that this approach can yield fourth-generation dendrons within 2 days without any significant defects in their structure [96]. Kothari et al. (2013) demonstrated an efficient and accelerated preparation of multifunctional dendrimers via nucleophilic substitution and orthogonal thiol-ene reactions. In this study, multifunctional dendrimers synthesized via an orthogonal approach did not possess the ability to carry out protection-deprotection steps to apply for complex organic molecule construction [97]. Moreover, the heterofunctional method is employed to produce dendrimers with a precise and large number of unique functional groups while maintaining structural integrity within the framework [98]. Goodwin and colleagues synthesized a heterobifunctional, biodegradable dendrimer through the creation of a symmetric aliphatic ester dendrimer derived from 2, 2-bis(hydroxymethyl) propanoic acid. The dendrimer featured a cyclic carbonate periphery and functional amine at the opening of the carbonate moieties. This approach was deemed advantageous for the formation of a dendrimer with eight alkynes and eight protected aldehydes on its periphery, as the resulting dendrimer did not require purification through chromatography [99]. All the above-mentioned methods require purification approach to yield pure dendrimer, either one way or the other. Hence, one pot synthesis has been introduced for the formation of dendrimers, which does not require any purification steps [100]. Recently, Yan et al. (2022) utilized one pot periodate oxidation approach for the preparation of dialdehyde cellulose and are modified with polyamidoamine (PAMAM) dendrimer of zero generation via hyperbranched crosslinking synthesis to yield cellulose-based dialdehyde polymers. The study showed that the dendrimer was grafted onto the backbone of cellulose with a crystallinity of 46.5%, uniform long-strip structure and excellent particle size distribution [101].

In recent times, several novel synthesis approaches were introduced for the preparation of dendrimers. Mahdavijalal et al. (2023) prepared a PAMAM dendrimer that is anchored to tungsten disulfide (WS_2_) nano-sheets via a grafting approach. In this study, the stimulus-responsive polymer was prepared by suspending WS_2_, 2, 2′ azobisisobutyronitrile (AIBN) as the radical initiator and poly (N-vinyl caprolactam) (PNVCL) in ethanol under a nitrogen atmosphere, stirring was conducted at 65 °C in a paraffin bath that was temperature-regulated, lasting for 7 h. The resultant surface-modified nano-sheets were utilized for the fabrication of three generations of PAMAM dendrimers via grafting, precipitation, and centrifugation technique. The electron micrograph results revealed that before the surface modification, the nano-sheets were 20–50 nm in size and had an inconsistent thickness. However, after the surface modification using PAMAM dendrimers, the size of the nano-sheets increased to 80–90 nm, with a more uniform thickness [102]. Further, Sohail et al. (2020) synthesized PAMAM dendrimers via a divergent approach with monomer coupling and monomer end-group transformation for the creation of reactive surface functionality as well as coupling of a novel monomer via solid-phase peptides or oligonucleotide synthesis. In this study, needle-like and spherical structures were produced during the synthesis of PAMAM dendrimers with ester-terminated half-generation and amino-terminated full-generation, respectively with ~827 nm of hydrodynamic size [103]. Furthermore, Bafrooee et al. (2020) utilized the chemical co-precipitation method for carboxyl-terminated hyperbranched PAMAM dendrimer formation, that is grafted with superparamagnetic iron oxide nanoparticles in a core-shell structure. The study revealed that the synthesis process resulted in the formation of a dendrimer with an average of 5.5 generations and an average pore diameter of 11.83 nm. The size of the dendrimer was observed to be in the range of 20–75 nm, with a spherical shape [104].

## 4. Bioavailability of Janus Nanoparticles

Green chemistry aims to limit or reduce the use and/or creation of hazardous materials starting from the initial design process to production and finally to the application of a product. This promotes prevention over remediation for material sustainability and safety. The production of Janus nanoparticles is via green chemistry. This is because Janus nanoparticles are mostly made of biogenic materials, including both natural and synthetic biopolymers that are biodegradable and biocompatible. This leads to the development of bioavailable, sustainable, and eco-friendly materials, especially for biomedical applications, which can eventually help to address the toxicity issues of some available materials. Organic and soft materials are used to fabricate most biocompatible and biogenic Janus particles using e.g., single/double emulsion, microfluidic, co-jetting, solvent evaporation, polymerization [64]. In addition, many biocompatible synthetic biomaterials are used for Janus nanoparticles synthesis which includes the FDA-approved poly(lactic-co-glycolic acid) (PLGA), polycaprolactone (PCL), and poly(lactic acid) (PLA) for their pharmaceutical delivery applications. They are often been utilized for time-programmed single/dual drug delivery and release at dual-site with simultaneous/stimuli-dependent release of two incompatible drugs due to their difference in biodegradability [73].

Some studies have reported the use of Janus nanoparticles with high bio-distribution for intercellular transportation and cellular uptake. For instance, Shao and their team have developed rod-like magnetic nanosized silica mesoporous particles via the modified sol-gel approach. The results indicated that a more targeted drug delivery efficacy has been achieved with higher retention and accumulation at the tumor sites via varying endocytic pathways, leading to higher intracellular internalization and bioavailability [105]. Another study by Shao, et al. [106] also demonstrated the bioavailability of doxorubicin-loaded Janus nanocomposites by concentrating the accumulation of the drug at the targeted site of the liver tumor with high tumor cell endocytosis whilst offering ‘zero’ release of doxorubicin to the other surrounding normal cells and blood circulatory system.

Many studies have investigated the cytotoxicity of Janus nanoparticles, indicating their excellent biocompatibility, especially for clinical applications. Cao and team have demonstrated the cytotoxicity of dual-drug loaded Janus nanoparticles in HeLa cells and MDA-MB-231 cancer cell line via the colorimetric cell viability assay [71]. Results revealed more than 90% of cell viability even with such a high concentration of Janus nanoparticles ~500 µg/mL. Moreover, it showed a lower IC_50_ as compared to free drugs. Furthermore, Janus nanoparticles are capable to offer a lowered systematic toxicity with significant tumor growth inhibition as compared to other core-shell nanoparticles [107]. Based on the cytotoxic Sulforhodamine B assessment performed under both normoxic and hypoxic environments, Janus nanoparticles can selectively eliminate the hypoxic liver cancer cells with dose-dependent cytotoxicity without inhibiting the normal surrounding cells for better and safer therapeutic outcomes. Besides, modifying the nanoparticles with silica could be crucial in enhancing their biocompatibility, specifically in the case of Janus nanoparticles.

Additionally, Zhao, et al. [108] have invented Janus hollow spheres with dose-dependent cytotoxicity. According to their research, the safety level of doxorubicin-loaded Janus hollow spheres was found to be favorable in the U87MG cell line when tested using the CCK-8 assay at both pH 6.4 and 7.4 with particle concentrations as high as 200 µg/mL. The results of both the cell viability tests and the pH-dependent toxicity evaluations indicate that Janus hollow spheres have the potential to be a secure and effective vehicle for targeted drug delivery. Another study has described the cytotoxicity evaluation of Janus nanoparticles after penetrating the blood-brain-barrier via the 3-(4,5-Dimethylthiazol-2-yl)-2,5-diphenyltetrazolium-bromide (MTT) assay using the rat C6 glioma cell line, at different time intervals and pH. The study revealed that the optimal conditions for maintaining cell viability above 50% were a minimum pH of 6.2 and a maximum incubation time of 24 h. Also, the results indicated that fewer Janus nanoparticles were present in non-targeted normal cells, likely due to the extended blood circulation time of the Janus nanoparticles, as well as the strong covalent bond between the encapsulated doxorubicin and its Janus compartment. As a result, doxorubicin was only released when triggered by the acidic condition of cancerous cells via breakage of imine bond, indicating the safe usage of Janus nanoparticles for targeted drug therapies.

## 5. Bioavailability of Dendrimers

**Figure 4:**
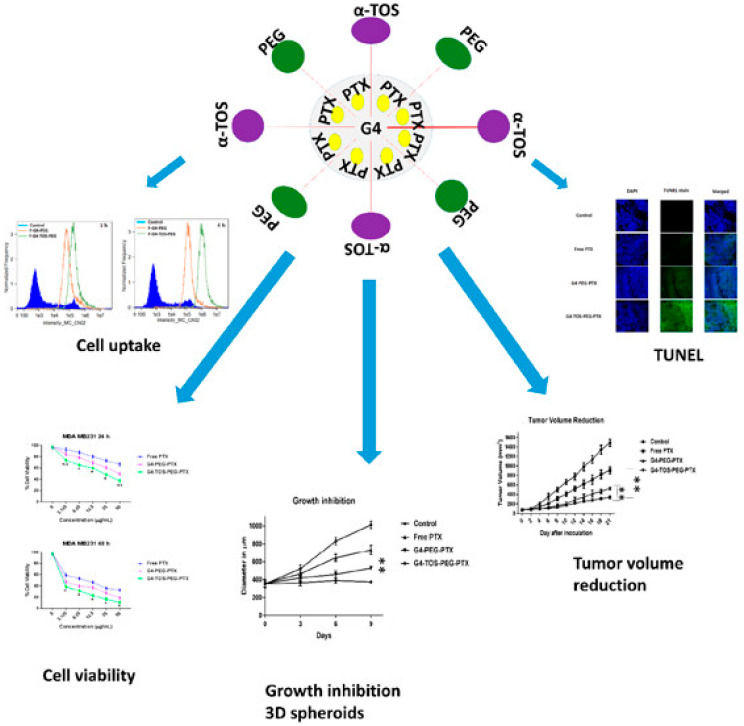
Schematic representation of dendrimers with PEG and alpha-TOS along with their cell uptake, cell viability, growth inhibitions of 3D spheroids, tumor volume reduction, and terminal deoxynucleotidyl transferase biotin-deoxyuridine triphosphate (UTP) nick end labeling (TUNEL) results. Reproduced with permission from Bhatt et al. Ref. [111]. Copyright 2019 ACS.

It can be noted that dendrimers are biocompatible and less toxic to be used in biomedical applications, similar to Janus particles. Rehman et al. (2021) prepared a novel resorcin arene-based dendrimer vesicles of nano-size based benzyloxy macrocycle. In this study, 4-benzyloxy benzaldehyde and resorcinol were dissolved in acetic acid under constant stirring conditions. Later, their pH was altered using sulphuric acid under reflux conditions for 24 h at 50 °C. A brown precipitate was formed as a resultant sample, which was filtered, washed with water, and dried, where the recrystallization of brown precipitate in tetrahydrofuran or methanol mixture (2:8, *v*/*v*) with 88% of yield. This study demonstrated that the synthesized dendrimer was highly effective for encapsulating quercetin, which was found to exhibit low toxicity in the NIH-3 T3 cell lines. The lower toxicity of the dendrimer-encapsulated quercetin towards cells is identified to be due to the existence of four hydrophobic benzyl groups with appropriate lipophilicity of benzyloxy macrocycle and their slightly amphiphilic nature with structural saturation [109]. Further, Giorgadze et al. (2020) synthesized PAMAM dendrimers of four generations to be useful for the encapsulation of silver atoms. The results showed that the dendrimers were stable at ~5 nm in size with strong visible light absorption. The study emphasized that the silver atoms in dendrimers can able to overcome the cell nucleus and membrane of 8–9 nm of pore size, which can subsequently reduce their cytotoxicity and improve their biocompatibility [110]. Furthermore, Bhatt et al. (2019) developed a dendrimer via alpha-tocopheryl succinate (alpha-TOS) conjugation and coating polyethylene glycol (PEG) on the surface of PAMAM dendrimer (four generations) as shown in Figure 4. The resultant dendrimer was utilized to improve intracellular paclitaxel delivery, which is a chemotherapeutic drug that is poorly soluble in water. The hemolysis assay revealed that the resultant dendrimer was nontoxic to red blood cells with high biocompatibility. Later, the cellular uptake assay conducted on B16F10 monolayer cells and MDA MB231 3D spheroids showed that the conjugation of alpha-TOS significantly improved the time-dependent uptake of the nanoscale dendrimer [111].

Noorin et al. (2021) created a novel second-generation linear globular dendrimer made of Gadoterate Meglumine and of nanoscale size.The resultant dendrimer was identified to be almost nontoxic towards immortalized human embryonic HEK 293 kidney cells and human glioblastoma cell culture [112]. Similarly, Ahmed et al. (2021) prepared a novel surface modified four generation PAMAM dendrimer via 4-nitrophenyl chloroformate as an activator and 2 kDa of PEG with spherical or semi-spherical morphology for the controlled delivery of anti-tuberculosis rifampicin drug. The findings indicated that the surface of the dendrimer was coated with PEG at a level of 38–100%, leading to a slower release rate of rifampicin compared to the non-PEGylated formulation and the unencapsulated drug. The study found that the fully functionalized dendrimer exhibited reduced toxicity towards raw 264.7 cell lines [113]. Likewise, Alfei et al. (2020) created a new biodegradable fifth-generation dendrimer based on polyester and featuring a free carboxylic group. The dendrimer was synthesized using dichloromethane, N, N’-dicyclohexylcarbodiimide (DCC), 4-(dimethylamino) pyridinium 4-toluene sulfonate and was protected with an acetonide group. Later, the dendrimer was used to encapsulate Etoposide (ETO), a substance derived from podophyllotoxin, the main chemical component found in the herbaceous plants named *Podophyllum hexandrum* and *P. peltatum*. The studies revealed that the dendrimer with ETO was 70 nm in size with nano-spherical morphology, 37% of drug loading, 53% of entrapment efficiency, and possess enhanced solubility in biocompatible solvents, such as ethanol and water. The cytotoxicity studies on neuroblastoma cells emphasized that the dendrimer and ETO possess cytotoxic and pro-oxidant properties and their bioactivity was synergistically improved after encapsulation due to the slow release of ETA [114].

## 6. Janus Nanoparticles for Target-Specific Delivery Applications

The advancement in nanotechnology has allowed Janus nanoparticles to serve as nanomedicines to regulate both the bio-distribution and tumor accumulation of administered medical drugs for better efficacy. Janus metallic mesoporous silica nanoparticles (JMMSNs) have been developed to be cancer-targeting for cancer theranostics with multi-functions such as magnetic resonance imaging as well as drug release and therapy [115]. This is attributed to JMMSNs that possess anisotropic compositions with stimuli-responsive properties to provide both tumor diagnosis and therapy synergistically [115]. JMMSNs are capable to load both diagnostic and therapeutic agents in separate compartments and release them spatiotemporally in response to different stimuli for the cancer-targeted delivery. Janus magnetic nanoparticles possess magnetic properties that make them suitable for both enhancing MRI imaging as a contrast agent and delivering drugs when used in conjunction with hyperthermia [116]. Hence, Janus magnetic nanoparticles are widely recognized as safe and effective carriers for biological drugs such as genes, proteins, and other biological molecules due to their controlled and sustained drug release profile and their ability to efficiently load drugs through the use of an external magnetic field. Moreover, the size of nanoparticles allows them to penetrate targeted cells as well as the blood-brain-barrier whilst maintaining the retention effect [117]. Janus nanoparticles have been revealed as a highly promising delivery vehicle for pharmaceuticals due to their superior properties, and they can be decorated with various targeting, diagnostic, and therapeutic biological molecules further to advance the use of nanomedicine with multiple functions. Table 1 shows a summary of reported works that exploited Janus nanoparticles for target-specific delivery and therapy.

## 7. Dendrimers for Target-Specific Delivery Applications

**Figure 5:**
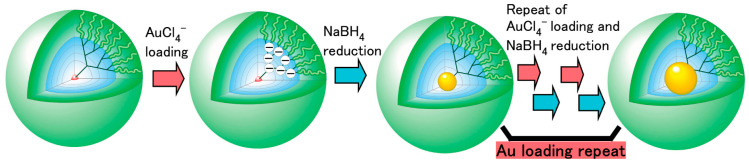
Growth of gold nanoparticles inside the PEGylated PAMAM dendrimers. Reproduced with permission from BUmeda et al. Ref. [122]. Copyright 2010 ACS.

Recently, numerous dendrimers were identified to be beneficial as potential targeted drug delivery systems for specific biomedical applications. Swanson et al. (2022) synthesized a novel fifth-generation PAMAM dendrimer, which was surface-modified and functionalized with folic acid. The remaining primary amines in the terminal dendrimer part were conjugated with the bifunctional 1, 4, 6, 10-tetraazacyclododecane-1, 4, 7, 10-tetraacetic acid, alpha-[5-isothiocyanato-2-methoxyphenyl]-, hydrochloride [9Cl] (NCS-DOTA) chelator, where stable gadolinium complexes were loaded into the dendrimer. The resultant dendrimer nanoparticle was about ~5 nm in size with the ability to be a potential target-specific magnetic resonance imaging (MRI) contrast agent with high affinity towards folate receptors in the liver and kidney for identifying human epithelial cancer cells in a murine model [120]. Similarly, Mbatha et al. (2019) prepared PAMAM dendrimer modified with folic acid and functionalized with nanosized gold particles for the efficient and targeted transport of exogenous small interfering ribose nucleic acid (siRNA). The study emphasized that the resultant dendrimer nanosized particles were spherical shaped with 65–128 nm of size and zeta potential above 25 mV, which indicates their high colloidal stability. Additionally, the dendrimer-based nanocomplex was less toxic to human embryo kidney (HEK-293) and HeLa-Tat-*Luc* cells (about 90% of cell viability). The study emphasized that the nanocomplex helped in the 75% increase in the induced transgene-silencing of siRNA and decrease the presence of excess folic acid for effective inhibition of hepatocellular carcinoma (HEPG2) and colon carcinoma (Caco-2) cells [121]. Likewise, Umeda et al. (2010) demonstrated the fabrication of polyethylene glycol (PEG) attached to a PAMAM dendrimer of four generations for the encapsulation of nanosized gold particles as shown in Figure 5. The results revealed that the nanosized gold particles were 2–3 nm in size with spherical morphology, which is engulfed by a single dendrimer particle, while the PEGylated dendrimer encapsulated gold nanoparticles were 15 nm in size. The study revealed that the dendrimers with gold nanoparticles possess enhanced toxicity against HeLa cells under the irradiation of visible light via photoinduced heat generation capability [122].

**Figure 6:**
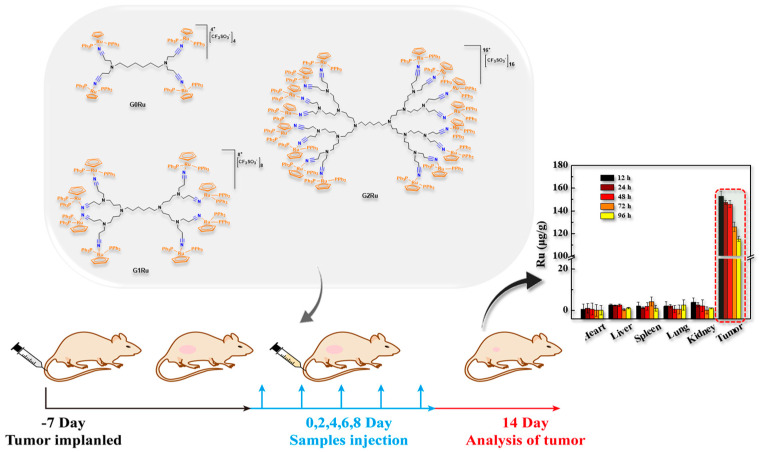
In vivo studies in an MCF-7 xenograft mouse model of low-generation (0–2) nitrile poly(alkylidenamine)-based ruthenium dendrimers. Reproduced with permission from Maciel et al. [127] Copyright 2022 Royal Society of Chemistry.

Xiong et al. (2019) exhibited the formation of carboxybetaine acrylamide zwitterion functionalized fifth-generation PAMAM dendrimer and morphine, which is an agent to target lysosome and to entrap nanosized gold particles. The study showed that the nanosized gold particles entrapped by dendrimer are beneficial for the delivery of the H1C1 gene, enhanced by serum. The results showed that the dendrimer-entrapped nanosized gold particles were of size ~1.5 nm, where the vector system helped to carry the H1C1 protein for effective cancer cell migration and metastasis inhibition [123]. Further, Sharma et al. (2021) prepared novel hydroxyl terminated PAMAM dendrimers, that are modified with sugar moieties, such as alpha-D-mannose, beta-D-glucose, or beta-D-galactose via click chemistry approach. The resultant dendrimers were ~4 nm of average particle size with nearly neutral zeta potential and are utilized to target tumor-associated macrophages and microglia. The study showed that the glucose-modified dendrimer has increased brain penetration and cellular internalization, compared to other sugar moieties. It has been proposed that these dendrimers can be potential delivery vehicles for the treatment of glioblastoma and other types of cancers [124]. Furthermore, Zhang et al. (2020) utilized in situ growth approach for the preparation of novel PAMAM dendrimers (0.5, 1.5, 2, and 2.5 generation) that are grafted on the persistent luminescence nanoparticles (PLNPs) surface. The aptamer AS1411 was coupled with the nanoparticle-dendrimer to specifically bind with the over-expressed nucleolin on the tumor cell membrane, thus increasing the intracellular accumulation of the nanoparticle. The PLNPs were spherical and 15.2 nm of average particle size. In addition, an anticancer drug named doxorubicin was also loaded in the dendrimer-nanoparticle-based delivery system via a pH-sensitive hydrazine, which was identified to be released in the targeted site of the intracellular acid environment. The new drug delivery system using dendrimers was found to have the capability of inducing apoptosis in HeLa tumor cells and inhibiting tumor growth [125]. The results of these studies suggest that dendrimers, particularly PAMAM dendrimers, have the potential to serve as effective drug delivery systems for treating specific diseases. Apart from drug delivery, dendrimers are also utilized as a potential gene delivery system. Ebrahimian et al. (2022) developed a novel lipo-polymeric PAMAM dendrimer-liposome, that are functionalized with transactivator of transcription (TAT) peptide and hyaluronic acid for targeted gene delivery system development. The study showed that the lipo-polymer possesses no significant toxicity with enhanced transfection efficiency in murine colon carcinoma cell line (C26), which will be beneficial for gene delivery applications [126]. Additionally, the incorporation or encapsulation of metals and metallocomplexes within the dendritic scaffold (metallodendrimers) holds the potential to yield novel metallodrugs or offer a new approach for the in situ delivery of metallodrugs. In this area, several works with in vitro and in vivo results were published by Rodrigues and coworkers, involving ruthenium compounds (an alternative metal for platinum-based anticancer resistant drugs) (Figure 6) [127,128], as well as platinum and platinum derivatives as anticancer drugs [128,129]. In any case, the research of the dendrimers is not exclusively restricted to the preparation of drug delivery systems. For instance, Rodrigues and coworkers developed new anionic poly (alkylideneamine) dendrimers until generation 3 with carboxylate and sulfonate terminal groups. These dendrimers have shown in vivo, to be very effective as microbicide against HIV-1 infection [130]. Thus, novel dendrimer structures were developed by the team, where their ability for targeted and controlled drug delivery is expected to be improved in the future.

## 8. Janus-Dendrimer Particles in Target-Specific Delivery Applications

**Figure 7:**
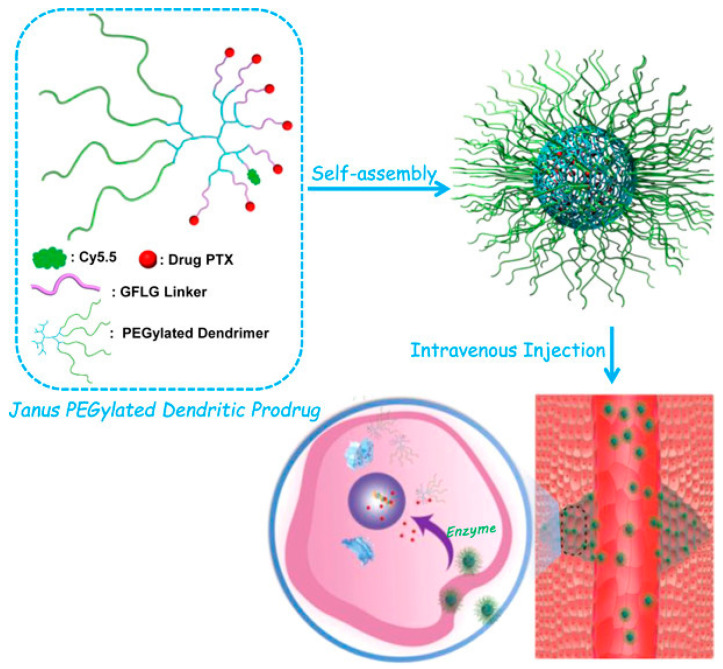
Schematic representation of PEGylated Janus-dendrimer and their self-assembly for intravenous injection in murine models for enhanced stability and anticancer efficacy. Reproduced with permission from Li et al. Ref. [135]. Copyright 2017 ACS.

The enhanced targeted drug delivery ability of Janus structures and dendrimer nanoparticles has led to the emergence of novel Janus-dendrimer nanoparticles to improve their controlled delivery. Further, Janus-dendrimer possess exclusive properties and structures in combinations with different end-group types and distinct surfaces, which makes them a better candidate to form unique drug complexes and conjugates [131]. These Janus-dendrimer particles were fabricated based on chemo-selective coupling, heterogenous double exponential growth, and mixed modular approach to be useful for stochastic and multiple drug conjugation-based combination therapy, solubility enhancement, antioxidant lyophilization, targeted delivery, and as fluorescent labels. In addition, the self-assembled Janus-dendrimer particles were identified to be beneficial for the vesicular delivery system, especially for pH-responsive delivery, targeted delivery, spatiotemporal delivery, and site-specific drug delivery [132]. Pan et al. (2012) created a new Janus-dendrimer particle that was amphiphilic and had peripheral groups made up of acidic amino acids and naproxen molecules, designed for efficient drug delivery to bones. The second-generation dendrimers with >95% of binding rates towards hydroxyapatite in bones, 28-fold enhanced the solubility of naproxen for about 5.37 mg/mL of concentration, compared to the standalone drug. Moreover, the study also revealed that the Janus-dendrimers do not possess any significant cytotoxicity toward HEK293 cells [133]. Further, Iguarbe et al. (2019) created an effective liquid crystal Janus-dendrimer particle made up of mesogenic blocks based on two third-generation Percec-type dendrons with terminal dodecyloxy alkyl chains and one or two carbazole units serving as the electrically active component. The study reported that the carbazole dendrimers were prepared via electrodeposition to form semi-globular particles with electro-polymerizable units. Also, the resultant particles were able to retain the rigid or flexible characteristics of the linker, which eventually influences the size of the particle [134]. Furthermore, a link was established between hydrophobic paclitaxel and the Janus PEGylated peptide dendrimer by Li et al. (2017), through the use of an enzyme-sensitive glycylphenylalanylleucylglycine tetrapeptide as a connector, using an efficient click reaction as shown in Figure 7. The resultant Janus-dendrimer particle possesses the ability to encapsulate 21% of paclitaxel with an average hydrodynamic size of ~69 nm, a narrow polydispersity index of 0.23, the zeta potential of −16.9 mV and spherical morphology. The study showed that the Janus-dendrimer nanoparticle release paclitaxel via enzyme responsive feature and is identified to be highly cytotoxic towards 4T1 (murine breast) cancer cells without any toxicity against normal cells, such as 3T3 murine fibroblast and C2C12 murine myoblast cell lines [135].

Recently, Falanga et al. (2021) created a new type of Janus-like dendrimer that incorporates peptides derived from the glycoproteins (gH and gB) of Herpes Simplex Virus Type 1 (HSV1), aimed at inhibiting viral infection. This was achieved through the combination of copper-catalyzed bio-orthogonal 1,3-dipolar azide/alkyne cycloaddition and photoinitiated thiol-ene coupling, producing both monofunctional and bifunctional peptidodendrimer conjugates. The study revealed that the peptides released by the formulation possess enhanced antiviral activity by inhibiting the DNA replication of HSV1, compared to conventional antiviral drugs, such as foscarnet, acyclovir, and cifofovir [136]. Similarly, Najafi et al. (2020) prepared a novel poly (propyleneimine) (PPI) dendrimer of the fifth generation with a core of cystamine and a hydrophobic surface. Later, the structure scission approach was used to convert disulfide bonds to thiol group and hydrophilic PAMAM dendrons were formed with amine end groups. The study demonstrated that the Janus-like dendrimer, with an average hydrodynamic size of 4.2–28.2 nm, has the ability to enhance the solubility of hydrophobic drugs such as dexamethasone and tetracycline [137]. Likewise, Zhang et al. (2022) designed a hydrophobic multifunctional sequence-defined ionizable amphiphilic Janus-dendrimer region via dissimilar alkyl lengths. The research found that the Janus-dendrimer particles greatly enhanced the activity of the hydrophobic 3, 5-, 3, 4-, and 3,4, 5-substituted phenolic acids they encapsulated, by up to 90.2 times [138]. All these studies showed that the Janus-dendrimer nanoparticles possess enhanced drug delivery capacity, compared to standalone Janus particles and dendrimers.

## 9. Limitations and Future Perspective

Dendrimers, Janus particles, and Janus-dendrimer nanoparticles have been identified as potential candidates for improved drug delivery applications. However, there remain several limitations that must be overcome before they can be utilized in large-scale commercial applications. The synthesis of Janus particles is one of the major limitations, as it is a multi-step process that can be tedious and challenging to achieve sub-micrometer particle size [139]. The various synthesis approaches, including masking, phase separation, microfluidics, E-jetting, emulsion, and self-assembly, each have their own drawbacks which hinder their scalability. Additionally, general limitations of nanoparticles such as polydispersity, size-dependent toxicity, and the use of hazardous chemicals in synthesis are also applicable to Janus nanoparticles [140]. Dendrimers also face several limitations, particularly at generations higher than 3–4, including high cost of synthesis, a lack of understanding of their effects on biochemical pathways and toxicity, and difficulties in engineering multifunctional dendrimers, especially the tediousness in purification and the yields of the end product. The toxicity of dendrimers has been reported in various studies and can result from improper processing, leading to undesirable side effects, low tolerability, and inefficient drug delivery [141,142,143]. To address these limitations, the use of lower dendrimers generations with less toxic terminal groups and the use of computational software and machine learning approaches have been employed to optimize Janus, dendrimer, and Janus-dendrimer synthesis parameters. These computational approaches have the potential to reduce the number of experiments and the cost of production, while providing greater insight into the interaction of nanoparticles with biological environments. However, these computational approaches are still in the early stages of research, are highly dependent of the data availability, particularly with regards to the synthesis of nanoparticles and evaluation of their properties and interactions in biological environments [144,145]. In conclusion, the future of Janus and dendrimer nanoparticles for improved drug delivery will likely benefit from a combination of enhanced electron microscopes, synthesis techniques, computational approaches, and machine learning techniques. This will enable the synthesis of novel and highly efficient nanoparticles with improved bioavailability for drug delivery applications.

## 10. Conclusions

Recently, there has been an increasing interest in developing a novel drug delivery system that can enhance targeted and controlled drug delivery while reducing toxicity and increasing biocompatibility. Janus and dendrimer particles have been introduced as potential drug delivery systems due to their unique properties compared to conventional delivery systems. These particles have the ability to encapsulate and release drugs in a controlled manner, acts as a drug per se, which can improve the efficacy of drugs and reduce their side effects. However, there are limitations to the use of these materials in commercial pharmaceutics, including lack of scalability, high production cost, and the use of toxic chemicals in synthesis. The emergence of Janus-dendrimer particles holds promise for overcoming these limitations. The combination of Janus and dendrimer particles can result in enhanced drug delivery and improved biocompatibility. However, it is important to improve the stability and toxicity of these particles in the future. This can be achieved through simulation, modeling-based computational and machine-learning approaches, as well as by optimizing the synthesis process and reducing the use of toxic chemicals. With these advancements, Janus-dendrimer particles could become a key tool in advancing drug delivery technology and improving patient outcomes. Their utility extends to various applications within the healthcare field and beyond, opening up new possibilities for innovation.

## Figures and Tables

**Table 1:** A summary of reported studies on the targeted delivery applications of Janus nanoparticles.

Janus Nanoparticles	Fabrication Technique	Target-Specific Delivery Applications	References
Anti-epithelial cell adhesion molecule (EpCAM)-coupled JMMSNs	Modified sol-gel method	To detect the circulating tumor cells and MCF-7 breast cancer cells in both blood samples and spiked cells with the use of magnetic-fluorescent nanoprobes. Rod-shaped EpCAM-coupled JMMSNs are capable of improving both the capture and binding efficiency of circulating tumor cells that have escaped from the site of the tumor. Anti-EpCAM serves as a targeting element whilst fluorescent probe loading allows bioimaging of the tumor cells.	[118]
Doxorubicin-loaded Magnetic Fe_3_O_4_- mesoporous SiO_2_ Janus nanoparticles	High-temperature hydrolysis reaction and modified sol-gel process	It is a multifunctional Janus nanocomposite developed to target and treat liver cancer by inhibiting tumor cell growth. The tumor-targeted drug delivery is accomplished through magnetic targeting and controlled drug release, resulting in improved permeability and retention. This helps to enhance the chemotherapeutic effects whilst minimizing the side effects via the magnetic-guided accumulation at the targeted cancer cells.	[106,119]
Stimuli-responsive Doxorubicin and 6-mercaptopurine dual-drug loaded Janus nanoparticles	Sol-gel method and modified Pickering emulsion technique	This Janus nanoparticle design enables real-time monitoring of dual-drug controlled drug release within living cells through the use of surface-enhanced Raman scattering and Fluorescence resonance energy transfer. The release of both drugs is triggered by the overexpressed glutathione and acidic environment of tumor sites, leading to better anti-cancer therapeutic effects as compared to single-drug loaded Janus nanoparticles.	[71]
pH-responsive Tirapazamine drug-loaded Janus nanocomposites	Sol-gel method	To treat liver cancer specifically targeting hypoxic over normoxic liver cancer cells, in response to acid stimuli. It works as a hypoxia-directed chemotherapy to enhance the synergistic radio-chemo-photothermal therapy that suppresses the growth of hypoxic tumor cells via enhanced folic acid-mediated endocytosis.	[107]
Bi-layered, pH-responsive PCL- poly(diethlyaminoethylmethacrylate) (PDEAEMA) polymeric Janus hollow spheres	Dual-step polymerization using silica particles: surface-initiated ring-opening polymerization and atom transfer radical polymerization techniques	A pH-dependent controlled release of encapsulated Doxorubicin at the targeted tumor site can be achieved. The result reveals a higher intracellular uptake and drug release rate of these doxorubicin-loaded PCL-PDEAEMA Janus particles at the pH of the tumor environment as compared to the physiological pH of other normal cells.	[108]
Doxorubicin-loaded folic acid-coupled Janus nanoparticles	Pickering emulsion technique	A potential SPION-based pH-sensitive targeted multifunctional Janus nanoparticles have demonstrated the capability to pass through the blood-brain barrier due to their surfactant properties for targeting and treating brain cancerous cells. Results show that Janus nanoparticles can be delivered specifically to the glioma tumor site with increased tumor accumulation using folic acid as the targeting element. In addition, the release of doxorubicin is highly dependent on the acidic condition of tumor cells with improved cytotoxicity.	[117]

## Data Availability

Data sharing not applicable. No new data were created or analyzed in this study. Data sharing is not applicable to this article.
